# Traditional Sports and Games: Intercultural Dialog, Sustainability, and Empowerment

**DOI:** 10.3389/fpsyg.2020.590301

**Published:** 2021-01-22

**Authors:** Soraia Chung Saura, Ana Cristina Zimmermann

**Affiliations:** Center for Cultural Studies on the Human Movement, Department of Pedagogy of the Human Body Movement, School of Physical Education and Sport, University of São Paulo, São Paulo, Brazil

**Keywords:** traditional sports and games, phenomenology, philosophy, festival, dialog

## Abstract

From Traditional Sports and Games (TSG) we have not only learned different ways of living time as well as inhabit space and a particular mode of practicing sports and games from distinct cultures, but also promoting universal dialog among people. TSG presents sustainable and ecological references for living needed even before the advent of the COVID-19 pandemic. Nowadays, environmentally friendly policies and production methods must be taken more seriously. TSG may reveal a path to sustainable development, considering our corporeality and cultural diversity. TSG are expressions of human groups that historically reproduce their way of life-based on modes of social cooperation and specific forms of relationship with nature, traditionally characterized by sustained environmental management. The purpose of this article is to discuss how TSG promotes intercultural dialog with a focus on sustainability, and how it empowers people and creates equality among its players. We understand that TSG can break socio-cultural barriers. For this study, we considered data from a Brazilian experience of TSG’s Festival held at a public school in the city of São Paulo (Brazil), organized in collaboration with our study group. Data consists of observations recorded in pictures and films during the processes of organization, preparation, implementation, and evaluation of a TSG Festival, held in a public school in São Paulo, Brazil from the years of 2017 and 2018, with the participation of 800 students from the first to the ninth grade of elementary school, aged between 7 and 17 years. The first step in our analysis is taken from a dynamic called “Talking Circles,” where researchers registered dialog about experiences and used specific literature about TSG, from a philosophical perspective. The team and students from our study group that organized these events were invited to participate in four different Talking Circles. Approximately 20 people participated in each one of these meetings. Recurrences that emerged from these Talking Circles are presented in the results and explored afterward. What does this experience–from bodies in movement, artistic or sporting, or both–teach about intercultural dialog and empowerment? Such gestures indicate a cultural heritage and corporeal wisdom that allows humans to face new encounters and understanding in peace, recognizing humanity common to all of us, regardless of our origins. Ethical and aesthetical results of such dialog reveal possibilities to be explored in our relationship with different cultures and the environment, providing points of sustainable development through TSG.

## Introduction

Since we all play together, Traditional cultural expressions like Traditional Sports and Games (TSG) demonstrate prominence in the ethical and aesthetic dimensions considering the fluidity and relevance of the groups in the elaboration of affects. Important research studies have been carried out about the diversity and richness of knowledge and learning provided by TSG all over the world (Parlebas, 2002, 2010; Lavega, 2004; Eichberg and Nygaard, 2009b; Gómez et al., 2012; Marin and Stein, 2015; Renson, 2016; Young Lee, 2016; Lavega-Burgués et al., 2020). For the scope of this paper, we understand TSG as a traditional community’s expression based on research and dialogs with Central and Latin America, especially in Colombia and Mexico, which have traditional games well consolidated both in academic studies and public policies (Herrera Velásquez et al., 2018). Based on this assumption, the purpose of this article is to discuss how TSG promotes intercultural dialogs with a focus on sustainability and how it empowers people and promotes equality among its players. Along these lines, we begin by defining traditional communities and their expressions, taking into account the Brazilian experience, and their current contributions. We would like to highlight the role of events and festivals in these communities, and then present the Brazilian case of a TSG Festival, held every year since 2017 at a public elementary school in São Paulo.

Observations recorded in pictures and films during the processes of organization, preparation, implementation, and evaluation of this TSG festival help us to discuss some fundamental elements present in bodily manifestations of traditional communities in Brazil. After the festival, we conducted four Talking Circles as a qualitative methodology to identify the recurrences observed by the researchers. Through them, we verified what seems to be most significant aspect for each person, seeking for more universal aspects. For humanities research, recurrences are indicators of meaning and potency (Bachelard, 2008) and guide the elements for the analysis. Our reflections were driven by the following question: what does this experience–from bodies in movement, whether artistic or sporting, or both–teach about intercultural dialogs and empowerment? Hence, the article focuses on the contribution of TSG for the discussion of three issues: intercultural dialog, sustainability, and empowerment.

## Traditional Communities in Brazil and Its Festivals

Brazil, with continental dimensions, holds a diversity of communities preserved, to some degree, due to the vast proportions of the country. These communities historically reproduce their way of life-based on modes of social cooperation and specific forms of relationship with nature, traditionally characterized by sustained management of the environment. Even in different habitats, such as large cities, we could verify that TSG maintains the expressions of such way of life.

Tião Carvalho, a master of traditional Brazilian knowledge, says this about the values he tries to spread in his classes and festivals: “These values, which are actually very old, are new to people in the city” (testimony in Saura, 2008). He is talking about the purpose of the festivals he promotes in São Paulo city. His festivals include plays and dances which originate from remote northern areas of the country. For about 20 years, Tião Carvalho has been organizing these festivities in the largest population center in Brazil, São Paulo State, situated in the rich southeast region. Although the festivities are eccentric for most of the city’s population due to its popular religiosity and original themes, each event brings together millions of people. Why does this festival, coming from a particular and exotic context, seduce so many people in the metropolis?

“Faith in the festivities, faith in the encounter,” explains Mr. Tião, showing that TSG expressions are traditional but also contemporary and alive today. Here we try to clarify how TSG operates with principles of collectivity, bringing together generations, valuing diversity, integration, and respect for the environment. These elements present different values from the valued individualism and meritocracy encouraged in multiple and complex ways nowadays, especially in large urban centers.

Lévi-Strauss (2013) reflected on the loss of reference that “Western type civilization” suffers: “Long an act of faith, the belief in a material and moral progress destined to go on forever is facing its gravest crisis. Western-style civilization has lost sight of the model it had set up for itself and is no longer bold enough to offer that model to others. Is it not therefore fitting to look elsewhere, to broaden the traditional frameworks to which our reflections on the human condition have been restricted? Ought we not to integrate social experiments that are more varied, more different from our own than those within the narrow horizon to which we have long confined ourselves?” (p. 4–5).

The ancient values derived from traditional expressions reflect a way of life that we are invited to look at, since traditional societies have been inhabiting the planet for 99% of the time of human life on earth, whereas, in contrast, our society as we know it today, 1% of this time (Lévi-Strauss, 2013). Therefore, our contemporary civilizations are exceptions as a reference of possible human existence.

By traditional community we mean: “culturally differentiated human groups that historically reproduce their way of life, relatively isolated, based on modes of social cooperation and specific forms of relationships with nature, traditionally characterized by the sustainable management of the environment” (Diegues, 2000, p. 22). This notion refers to indigenous populations and Afro-descendant communities (*quilombos*), among others, in an intense relationship with the environment. In the case of Brazil specifically, where exuberant natural areas are still preserved, there is a rich diversity of these communities, more or less isolated. They maintain their respective ways of life despite the impositions of the Western capitalist system. Here, at least 15 different human groups were identified. Each group has its particularities in their way of life, as well as in terms of TSG. Taking a group of Afro-descendants as an example, there are about 700 *quilombos* that practice capoeira regularly today. Capoeira, a dance influenced by martial arts, acts as an expression that reflects the group’s identity and recalls the still recent struggle for freedom (Saura, 2019). These populations steadfastly protect their environment and habitat, living in complete interdependence with the environment, practicing close observation, and especially, by the way, they prevent external factors from threatening their areas (Brasil Ministério do Meio Ambiente, 2000). Therefore, we are talking about different ways of understanding and acting in the world. Contrary to the misconception that traditional is stagnant and unchangeable, these communities show us how knowledge and tradition are always being updated. However, they maintain certain fundamental structures (Saura and Zimmermann, 2016). According to Cunha (2007): “There are at least as many traditional knowledge regimes as there are peoples. For while there is, by hypothesis, a single regime for scientific knowledge, there is a legion of traditional knowledge regimes. There is no doubt, however, that scientific knowledge is hegemonic. Modern hegemonic science uses concepts, traditional science uses perceptions” (p. 79).

As in science, knowledge is always being built on the process of constant update. However, although all knowledge rests on the same logical operations and responds to the same thirst for learning and understanding the world around us (Lévi-Strauss, 2013), there is this significant difference between them: concepts and perception. Knowledge and other expressions from traditional communities reveals a learning process that necessarily passes through the human body and the senses. It is worth calling attention to the fact that traditional practices of building knowledge can perceive and anticipate discoveries validated afterward by hegemonic science due to the ability to understand the context, the interrelations and the circumstances. Their historical relationship and observation of all the phenomena made them discover and anticipate issues for science, as we have seen in biology, pharmacology, preservation, and reproduction of species, and even in social sciences, by the way many of these societies are organized. The accurate observation of nature places these populations in a prominent spot in the production of knowledge, and in the symbolic and mythical system that makes up the repertoire of humanity since ancient times (Bachelard, 2008). For these populations, the environment has been part of their symbolic system and appears on TSG expressions. This way, TSG reveals and updates the fundamental images and values from these populations, as master Tião Carvalho reinforces. Hence, the notion of the environment is extended and inclusive: “The environment came to include not only the environment of humans, but the environment of all life forms. Life then came to include more than living things encompassing rivers, landscapes, cultures and ecosystems. One started to talk about the living earth” (Breivik, 2019, p. 65).

Clearly we can see how TSG is related to the environment, culture, history, and political struggles (Calegare et al., 2014). These practices, made up of simple elements and complex, human nature observation technology show us a path to intercultural dialog, sustainability, and empowerment. Very often, games take place on important dates and celebrations for each one of these communities. The festivals are events that reflect the symbolic capacity of humankind to give meaning to things and events from what one observes. These festivities range from birth celebrations, hunting rituals, harvest parties, thanks for abundance and food, to other manifestations concerning human observations of the movement of nature, seasons, and life. They are translated into everything produced for these celebrations: body props and space, music and dance, food, rituals, narratives, artistic and craft productions. They go back to times of mystery, to the active human with creative imagination–this ability that, in addition to logic and reason, allows us to create, invent and assume the impossible (Durand, 2012).

As many of these celebrations end up with a race or a game where everyone participates, TSG plays a role in these systems. The festivities promote bodily engagement (Merleau-Ponty, 1962) of the whole community, concentrated in a single event. The notions of dialog and sharing are enriched by the experience of intercoporeality (Coelho Junior, 2003). Celebrations and festivities are present among all peoples and nations and help us all to align values and develop new meanings for our daily practices. These perspectives show different possibilities of being with others and introduce new forms of existence. And as Mr. Tião reminds us, presenting something new is not necessary. It is important to note that the recognition of interdependence relations is notably directed at personal relationships. In light of a traditional community’s way of living and expressions, we prefer to align our discussion with a less anthropocentric perspective and more ecocentric premises.

## Methodology

For this study we used data from TSG Festivals organized by the study group PULA, from the Center of Sociocultural Studies at the School of Physical Education and Sport/University of São Paulo/Brazil, in collaboration with a public school from the city of São Paulo, the EMEF Desembargador Amorim Lima. The “TSG and Street Leisure Festival” has been running since 2017 and engages a whole primary school, including students, teachers, and staff. Data for this article consists of discussions from observations registered in fieldwork during the process of organization, preparation, implementation, and evaluation of the TSG festival from the years of 2017 and 2018. For data collecting, we organized four Talking Circles after the festivals coordinated by two professors in each edition of the festivals. In the first and second meetings, ten researchers involved in the Festival identified issues related to the Festival itself. In the third meeting, we listened to the students from two Physical Education (PE) undergraduate disciplines, which participated in the festival. And in the last meeting, we brought the findings to the research group, so we could identify the recurring themes that emerged from both groups. Talking Circles is a proven methodology that has been applied in traditional communities works, and integrates academic research with traditional populations and native peoples (Tachine et al., 2016).

This annual festival lasts an entire day and is attended by students from this public school that has a partnership with our university. In total, about four hundred students attend in the morning and another four hundred students in the afternoon. Students are from the first to the ninth grade of elementary school, aged between 7 and 17 years. Students from this school reflect the socio-economic and ethnic heterogeneity present in the surrounding neighborhood. Although all social classes are represented in this school, a predominance of families are from lower income levels. This heterogeneity is highly enriching from the perspective of solidarity. According to the school’s evaluation, students from families with very different income levels establish friendships and bonds that favor exchanging life experiences.

At each event, games and activities from childhood culture are selected. A commonality to these games is the fact that they are strongly present in the traditional communities in which we work. The festival takes place in the street, considered to be a public space. Every step of the process requires pre-planning. It is extremely important to consider TSG’s background to organize a festival properly. There are many challenges presented by contemporary societal dynamics, and it is essential to consider previous experiences and research to support educational perspectives (Eichberg, 2009).

The first step was to start planning with the school EMEF Desembargador Amorim Lima. We held meetings, one for each event, with the Head of School, teachers, and staff at the beginning of the school semester. The school is located close to our university campus, making it easier to work with undergrad students. Together with the school’s team, we discussed which games will be played and equipment needed. Games that do not require major material investments were given preference. These meetings also helped teachers prepare the students for the event. During the school year, students researched and played traditional games. In parallel, we trained PE students to assist teachers and students during the festival.

Activities at the festival include jump rope, spinning, marbles, stretch games, “peteca,” hula hoop, and yo-yo, among others. We consider that one important note–learned from other Festivals initiatives–are gestural inspirations provided by experts in these games. Thus, at each event, there are special guests, experts in one or other game as yo-yo or top, who always dazzle children. The choice for using a public space in front of the school encourages neighborhood participation. It reinforces the importance of valuing the public character of streets and sidewalks. The street in front of the school is closed for cars, creating a safe environment for playing.

During the festival, undergraduate students, researchers, and schoolteachers stand at different points on the street, according to the game they’re participating in. Children can move freely without space or time limits, without age, gender or grade restriction. For everyone, according to the reports collected, the day is over in the blink of an eye. Teachers and PE training teachers were available to help students with materials when needed. The idea is to leave the students free to choose what they want the most, relating to the use of materials, the place, and the time available.

This study has a qualitative approach, considering its characteristics and theoretical references. Talking Circles proved to be the most appropriate methodology for identifying recurring themes among what researchers and undergraduate students considered most significant in the Festivals. Talking circles started with us all sharing audiovisual materials from the festivals. Each group–undergrad students, schoolteachers, and researchers–took pictures focusing on relationships, recurrences, or anything else notable. For this study, we selected images with a focus on relationships between the participants. After sharing and selecting audiovisual records, direct questions were asked to encourage discussion. Teachers, researchers and undergrad students at the festival commented on issues involving intercultural dialogs, sustainability and empowerment. Definitions about these issues considering the urban context were previously made by the conductors, and presented at the “Results” section.

Approximately 20 people from the research group participated in Taking Circle. Participants were free to talk about subjects that they considered to be relevant in the experience of promoting the Festival and observing the children’s engagement. Recurrences considered for discussion in this work were mentioned more than five times in the Talking Circles.

The search for recurrences–what is repeated everywhere regardless of the cultural environment in which people are present–is part of this methodological approach. This research attempts to investigate subjectivities. Such perspective presupposes considering the first person perspective as the one who lives the experience. But the collection of this rich and human material presents itself not only as an individual or particular component, in this perspective it may bring traces of our human existence. Therefore, this approach emphasizes experience, or personal experience, but seeks among them what is true for every human being, to be able to make more general postulates and not just private or individual observations. In cultural terms it is not possible to generalize conclusions from one group to another but adopting a philosophical perspective it is possible to explore elements that belong to humanity (Martinková and Parry, 2011). In this sense, it is crucial to understand subjectivity and recognize its relevance in how knowledge is produced. It is methodologically significant specifically in the perception of human manifestations where the human being cannot be studied only as an object, considering the complexity of the phenomena. Talking Circles as a methodology requires attentive listening to promote a horizontal relationship between professors and students, aligned with traditional communities’ values. It harkens back to oral tradition, to horizontality, to listening more than talking, respecting diversity and understanding of one another (Tachine et al., 2016). Based on the principles of Paulo Freire (2015), Talking Circles was the moment to elaborate on everything that we saw, felt, and learned from the festivals. It was the moment to reflect on what enchanted us at TGS Festivals and why.

## Results

The team and students from our study group that organized these events were invited to participate in four different Talking Circles. Recurrences that emerged from these Talking Circles are presented below in the results and explored afterward. Triggering questions were: “What does this experience from bodies in movement, which updates children’s gestures in traditional games, promote among its players? What does enchanted us all on TSG Festivals, and why?”

The participants were surprised at the high level of engagement from the children and their teachers. Everyone in the Talking Circles noticed that cell phones were hardly used throughout the day. This engagement was a pleasant surprise, which showed the strength of TSGs even for children growing up in the city.

That led us to other inquiries related to intercultural dialog, sustainability and empowerment. Talking Circles focused on each one of these main themes. Specific and general questions were asked about the topics, as indicated in Table 1. Most participants gave similar answers and expressed surprise at the results overall and level of children’s engagement in particular.

**TABLE 1:** Themes, Questions, Indicators and Recurrences developed in the Talking Circles.

Central themes	Questions and Indicators	Recurrences
Intercultural dialog	How does the encounter between different cultures in the school arise from the game? What are the differences between the participants? Can interculturality observed in traditional communities be transferred to school? How? How do children appropriate body repertoire of games? How do they play together?	Gesture of traditional communities updated on children Play and do-together Adults and children playing together Boys and girls playing together Boys and girls of different ages playing together Boys and girls from different social classes and ethnicities playing together.
Sustainability	What attracts children to different games? How is to play in the street? Do they try different things in different spaces? How do they learn? Do they enjoy playing on the streets? How do they use the space available? Is there interaction with people outside the school? Do they demonstrate empathy with differences between them?	Materials and simple elements such as shuttlecocks, marbles, tires, ropes, rubber bands were fascinating. Body inspiration and gestural references are important. Use of public space. Environment interaction: playing with elements. Environment perception: playing in wider spaces Interaction with the community surrounding the school. Interaction with each other. Playing together.
Empowerment	Do children feel the desire to learn these games? Did the children engage in any of the possible games? Did the children demonstrate vibration and power? How did they resolve conflicts? Children who usually do not participate due to gender, race, disability? Are children who already know the game generous with each other? Children who want to play alone?	Integration Autonomy and spontaneity to choose which game they would like to engage in. Horizontality in relationships. Highlighted body awareness. Learning new bodily skills and gestures. Ethical presence: debates on how to play, joint development of rules and new challenges. Conflict resolution with innovation and autonomy. Disabled children playing together. Remember new gestures and body skills. You can play alone if you want and need. Play together.

*First question: After participating in the festival and seeing the images, what moved you?*

For this paper, we consider broad definitions of intercultural dialog, sustainability, and empowerment. These notions that are derived from fieldwork in traditional Brazilian communities bring other indirect questions to researchers when applied to an urban setting. For instance, in academic literature, intercultural dialogs refer to the dialogical encounter between subjects from different cultures. In the case of Traditional Games and Sports that occurs at these festivals in large urban centers, intercultural dialogs may be identified as bringing together children with different backgrounds playing together.

We have shown how corporeality favors this dialog between people from different backgrounds. Teachers witnessed how, throughout the year, children form cohesive groups and often do not mix. Those barriers were broken during the festival, where children were more interested in the games and activities, which encouraged them to play together. Analysis of materials collected from the Festivals shows that even seemingly watertight social barriers can be broken.

As an example, we can mention how teachers’ roles were reversed in many festival situations, as their students taught the teachers how to play. Similarly, in Talking Circles and by observing pictures and videos, the participants noticed the reversal of other structural differences. They identified that children of different ages, children and people from different social classes and ethnic groups, children with neighbors from different backgrounds, as well as adults, were are all playing together.

In urban environments, sustainability takes on a different meaning. They refer to using public spaces, encountering other people, the empathy promoted by playing together, and the dialog that is established with others. It refers to realizing that there is no need for expensive toys or large investments in material resources for playing games. Children who participate in TSG festivals learn to share spaces and simple equipment. They also learn to make their own toys from simple material. Expensive toys and the entire entertainment industry are unnecessary for us to be together, learn and have fun. Participants from the Talking Circle also noticed that there were many moments of interaction with the school community. Through games, gestures and rules, it is clear that traditional communities values, such as learning together, healthy and happy competition, are shared.

For this work, we consider empowerment as the appropriation of bodily knowledge established in relationship with the world. It is a concept that reveals a learning process that necessarily passes through the human body and the senses and emerges when we incorporate something new. It is an “I can” movement (Merleau-Ponty, 1962) that requires full presence and usually comes from a desire to achieve specific objectives–the player tries, insists and succeeds. During the Circles, many participants mentioned that some children were playing alone, but only until they learned the game’s techniques. After acquiring some new skills, they would find other children to play, teach, and show. At this point, repetition is part of this process, deepening the game structures in new ways. Freedom and spontaneity also seem essential in running the festival: children play with what and whom they wish. The goal of playing together was for the sake of the game and improvement.

Although we are not in one of the contexts of traditional Brazilian communities, it is necessary to understand how these communities’ values can be updated in the bodies that play. Besides, even if it was not the festival’s intention, as we can see below, “doing-together” stood out as a premise of freedom, potential, intercultural dialog, sustainability, and empowerment.

Certainly, doing together, one of the core values that touch traditional communities, was present in the three themes observed at the festivals. Masters of traditional knowledge often respond when asked how they teach: “I don’t teach, I do it together” (testimony by Tião Carvalho in Saura, 2008). Traditional communities consider the voice of the elderly like being the voice of the world’s past. This existential doing-together dialogs with traditional perceptual knowledge and happens without the need for words. This knowledge emphasizes the primacy of experience, gestural and corporal reference. Boaventura de Souza Santos refers to the primacy of the senses in the production of knowledge as inherent to the Epistemologies of the South (Santos, 2019). In his proposal, the “South” represents excluded, silenced and marginalized populations exploited by colonialism and capitalism. So, “the global South is not a geographical concept, even though the great majority of its populations live in countries of the Southern hemisphere” (Santos, 2016, p. 18). Doing-together is a recurrent *modus operandi* among these populations. Children and the elderly are included within the knowledge transmission system, which triggers our shared corporeality (Merleau-Ponty, 1962). Observing gestures from the more experienced players or creating technologies is part of a learning process for children. When they reach a particular manual skill level, they can follow the process. This bodily perspective appears in different possibilities of being with other people and introduces different ways of aligning values and developing new meanings for our daily practices. Doing-together requires trust, active participation and the understanding that being a child is a presence of now, and not only a promise of a future.

## Discussion

In the film “Promises” (Goldberg et al., 2001), Israeli and Palestinian children share the harsh reality of separation and hatred, despite being just 10 min away from each other. The cameraman interviews them to ask what they think of each other. They speak of themselves through past generations’ voices talking about war, the enemy, and anger. The echoes of many previous generations are in the children’s voice. When invited to a meeting with each other, they hesitate, but finally, they accept the documentary’s filmmaker idea. The proposal now is to establish a real communication between them, a body of communication in a playful situation. At that moment, ingrained resentments and hatreds are set aside. Children play together. Eichberg and Levinsen (2009a) also provide an example of the power of a Popular Movement Culture for conflict management. They explore the experience of a popular football festival, different from a competitive and standardized sport, in the Balkans. The festival was the first multi-ethnic event, including Muslim refugee children, to take place in Srebrenica after the civil war and brought children together through a common language. Similar examples are found elsewhere in the world.

This paper aims to discuss how TSG promotes intercultural dialogs with a focus on sustainability and how it may empower people taking into account the experience of TSG festivals in a public school in São Paulo–Brazil that attends 800 children from 7 to 17 years old. We analyzed some recurring elements that emerged from within the Talking Circles with the researchers from our study group and PE undergrad students who participated in TSG Festivals. Intercultural dialog, notions of sustainability, and empowerment through TSG were widely discussed. Some schoolteachers wrote to us gratefully, giving their testimonies of what they perceived during the festivals and later, on the impact of day-to-day activities with the children. These testimonies are not presented in this paper but reflect the interaction among all participants.

Here in this section, first we present our experience on TSG area, how we had inspiration for realizing a festival, and how recurrences of gestures called our attention. Considerations of the corporeal engagement and the importance of Festivals for children and for the city are taken. We highlight how the practice of traditional games may promote global health, adding elements of the main subjects: sustainability, intercultural dialog and empowerment. The originality of this session is to situate the TSG not as local event, but as bodily practices that dialog with the humankind, regardless of cultural origins, as they are located in our symbolic gestures and corporeality.

### The Festivals Gets Into the City–The Experience of a TSG Festival in São Paulo City

It has been a long way for us to understand TSG as an important phenomenon for thinking academic research and public policies. After conducting an International Seminar delimiting the field of knowledge with invited researchers from universities of Europe, Asia and North America (Saura and Zimmermann, 2016) our department turned to Latin and Central America researchers, understanding that among them “knowledge is embodied” (Santos, 2019, p. 136). In the event that followed, we received researchers from the University of Antioquia (Colombia) and from the Mexican Traditional and Autochthonous Games and Sports Federation (FMJDAT/Mexico), who presented their good academic practices and research experience. A year later, on Colombian soil, we witnessed the “Traditional Play from the streets” (JRTC) in Caldas, municipality of Antioquia. In this festival, which is in its 37th year, we saw an entire city in the streets to play for five consecutive days (Gómez et al., 2012). Conceived by Master José Humberto Gomez, the phenomenon inspired the Street Games Festivals in São Paulo, carried out by the PULA Study Group at the Sociocultural Studies Center-EEFE-USP. This background has been fundamental to support our actions and research on TSG.

The international community mentioned the same persistent gestural recurrences we see in TSG festivals in Brazil. Some materials, the ones that we see in traditional communities, encourage the same gestures even from school children, taking into account that many of them have never left the city. By the same gestures, we mean a familiarity of body behavior in dialog with similar provocations. The well-known work of Mauss (1979), first published in 1935, brings to light the cultural mark of body techniques. The anthropologist highlights the relationship between biology and culture registered as corporeality. From this, it may be assumed that even universal games, such as the spinning top, promote a cultural encounter. Through detailed observation, one might notice that some children use different fingers to hold the top or tie the beard in different ways to find a variety of results. However, there is a proximity of gestures that results from the relationship that the player establishes with the elements of the game. As Parlebas (2003) advises, it is important not to be tempted to reduce the game to the context’s characteristics or the player. The game has an intrinsic reality that is perceptive by traces of motor action. These motor behaviors are noticeable in the player’s relations with his environment: space, objects, time, and other players. From a phenomenological perspective (Merleau-Ponty, 1962), we understand that the materials and equipment suggest a corporeal engagement, like a language that puts us all in dialog sharing differences based on common soil. This is what can make the game a fruitful locus for bringing different cultures together. Through the body, we are invited to enter cultural and mythical aspects (Bachelard, 2008) in the same eco-sustainable postures that we frequently find in our research field. Also, despite its low cost, the high social impact of the material used to play is remarkable.

Considering the experience from the so called “TSG and Street Leisure Festival” and analysis from different fieldwork carried out over the last 10 years by the Pula Study Group (Saura and Zimmermann, 2016, Saura and Zimmermann, 2018), it is clear that traditional games actually preserve structural elements of human kind in magnificent reproductions of body images. A Greek painting depicts the movement of a boy playing a spinning top in the ancient age. Their moving image is the same as boys and girls in Brazil and the world today. Incredibly and without any apparent contact with each other, the same gestures appear. The scenes are repeated in schools, streets, alleys, in a game that historically has existed since ancient times, throwing with the same effusiveness arms and eyes of attentive boys and girls. During the Talking Circles, this was the first strong aspect that everyone noticed. “Children know how to play. Feels like they remember. They all forgot their cell phones when they saw a top. They were delighted. It was like responding to an inner call.” (Testimony in the Talking Circle). We speak of this place where the tradition is located in the gestures that children show us today–here and now–with archetypal elements that cross different times and spaces, from ages ago to the present day. TSG embodies the collective human memory. Game creators have already identified this potential. In the case of the spinning top, one can mention updates of the object that have become real “fevers” among children, not by chance, like the blay-blade and the spinner.

Groups understood games and activities as a kind of dialog (Zimmermann and Morgan, 2011). This dialogical condition is ethical since rules are created and recreated in intermittent debates (Bruhns, 2004), which above all require full presence in what children do. Moreover, one recurrence and even a surprising character was to perceive that the relationships among the festival participants were horizontal. PE Students expected to teach. In the beginning, they were unsure about our organization; they were in a much smaller number than the children. But some children knew how to play much better than our students. In TSG, there is no clear border between who teaches and who learns: facing the materials, everyone feels challenged and attracted by the game. They all notice that other common barriers were dissolved during these festivals: social status, age, and gender. In this case social barriers do not matter for playing since the same rules apply to everyone. Many mixed groups were seen playing together. Older participants may be a reference for how to do it, but there is not a hierarchy. PE Students also mentioned they do not perceive gender or age barriers, since the groups were mixed.

The educators are the children themselves, and the players are all. New elaborations appear on all sides, precious, surprising scenes. In fact, the objects surrounding the practice of TSG do not require significant investments and are like gunpowder, material provocateurs of this human repertoire, as Bachelard (2008) already reminded us. During the Festivals, students invite teachers to play together, some of them ask for help, while others teach their friends. Besides, different elaborations with the same materials were experienced. New groups of children from different ages were spontaneously organized, including students from inclusion programs with disabilities.

The spinners are set aside in front of a spinning top–firm strings between the fingers. A member of staff is willing to teach the game of his boyhood to children. A vibrant wheel is formed; the tops spin in the air, enigmatic, spectacular, flying. It can be seen that when a child plays, in a very authentic way, it strengthens ties with the most human of each of us.

The Talking Circles noticed that how the children organized into groups was always changing. The street becomes alive in a safe, playful environment and during two festival events no incidents were reported. The own children solved minor conflicts, and different solutions were found for everyone to play. Teachers reported that the project has helped to improve the repertoire of games and the students spontaneously chose TSG as a theme for a big annual festival at the school. Another interesting report was that after noticing that some children were having several relationship problems during their free time, the school made TSG materials available and the problems were over. Research conducted in a very different context showed promising results on the improvement of students’ relations and socialization in primary education after traditional games implementation (Kovačević and Opić, 2014).

For the teachers who conducted the activity, none of this was entirely new, as we mentioned before, there is Caldas, municipality of Antioquia, Colombia, where an entire city gets to the streets annually to play (Created by master José Humberto Gomez, the “Festival de Juegos Recreativos Tradicionales de La Calle” showed its expertise and inspired these similar events in São Paulo). At that festival, experienced practitioners were a reference for children and young people. Therefore, for these “TSG and Street Leisure Festival,” Anselmo Gomes, the Brazilian national yo-yo champion was present and left the children in amazement.

In a city where contact with childhood becomes increasingly reduced, where the landscape is increasingly “grown-up,” we see, less and less, boys and girls playing in public spaces. Since the festival breaks down school cliques and takes place in the surrounding streets, children and young people realize that the street is a place of possibilities, a common space for all of us.

Considering these investigations, it is currently believed that TSG should be part of public policies. Play is important not only for small children but for everyone. Moreover, TSG in PE and Sport enhances experiences, not only of classes and training but also of a whole relationship with the body, with space, the street, the school, with the diversity of the whole city at last. Research, fieldwork and interviews with partners reveal expressive bodies that are willing to play again.

### Intercultural Dialog, Sustainability, and Empowerment

According to United Nations Educational Scientific and Cultural Organization [UNESCO] (2015) TSG are considered an intangible heritage of humanity. UNESCO stresses, “the practice of traditional games promotes global health.” What seems to be the ethos of TSG is this bodily dialog, which reveals the humanity in all of us, regardless of geographical, social or cultural aspects. From this perspective, sports and games are a common language, spoken with a material call to action. Children are, for example, completely infatuated by the bow and arrow. It is a material that invites them to play. With the equipment, players repeat the same gestures and archetypal stances of warriors existing in all nations. We are talking from this understanding of the traditional as something located in the gestures that children show us today–in the here and now–with archetypal elements that cross different times and spaces. It is possible to find the same gestures in the traditional communities of Brazil (Hackerott et al., 2017). The game evokes, in the body, the very materiality of the arrow, with images of speed and straightness (Bachelard, 2008).

Moreover, similar gestures are found around the world. In these recurrences, we find something prior to the traditional nature-culture dichotomy. We see a gesture that is a precedent, which is characteristic of humankind. (For instance, the first records found of the top spinning game, a traditional children’s toy that turns on itself: a Greek painting of a boy playing the top in the old age). The image of the gesture of a child playing a top is the same as that performed by boys and girls today, whether in the interior of the Amazon rainforest, on the northeastern beaches, among Xingu Indians, in schools or on the outskirts of urban centers. It is a traditional game that dialogs with boys and girls from all historical times and without defined geography. So we have seen how TSG promotes intercultural dialog, coming from a knowledge that takes place in the corporeal experience of the gesture. Like knowledge developed in traditional communities, knowledge produced by TSG is of the order of perception. In Caldas, Dom Antônio manufactures spinning tops in broad daylight, surrounded by avid boys and girls. The master boasts of having trained the best players in the country. His tops are considered the best in the world, perfect and exact. When we see him perform with the children who grew up under his care and learning, we were all shocked. The tops dance with their players’ bodies, are launched into the air infinite times and never touch the ground. This hypnotic fly, turning around its own axis in space, continues to enchant, over and over, people from all over the world without causal explanations. Mastering this lively and aerial dance performed by the top is the desire of many players.

Durand(2012, p. 60), inspired by Merleau-Ponty, considers that “the whole body collaborates in the constitution of the image,” and that there is “close concomitance between body gestures, nerve centers and symbolic representations.” In this anthropological perspective, body language is also considered as a symbolic language, that is, of generating meaning for human existence, so perhaps the existential insistence on performing certain gestures.

Sports and games have been presented as a fruitful dialog for the culture of peace (Saura et al., 2018). Regardless of the complexity of its rules and the competitive content employed, the game’s dialogic character is one of its main characteristics (Zimmermann and Morgan, 2011). This dialogical character protects ethical possibilities to the play. The game requires players to be fully present. In the game, the impediments of age, gender, space, those who teach or learn, languages, social classes, and different cultures are mitigated. The knowledge that the game requires is based on corporeal experiences (Zimmermann and Saura, 2016).

It is also an aesthetic issue, as the game comes from this perceptual, subjective knowledge, the bodily experience about which it is not always possible to explain. That is why the theme was chosen by United Nations Educational Scientific and Cultural Organization [UNESCO] (2015) as one of its priorities for action. It was also selected as an activity to bring together a divided country, such as Korea. For UNESCO TSG practice “promotes global health,” placing players in an intersection between past and future, reaffirming the specific identity of peoples in the age of globalization, while their recurrences and similarities reveal certain thematic that are universals. It is perhaps in this way that games promote peace: through intercultural and corporal dialog that can be established when these themes are in action, that is, in the very act of playing (United Nations Educational Scientific and Cultural Organization [UNESCO], 2015).

Considering elements from fieldwork and references from different cultures we may understand that TSG bears the mark of many cultures and connects with humanity that which permeates us all. It is possible to observe an approximation of gestures in similar games, similar ways of establishing relationships with the environment and the relationship of proximity with organic elements, offered by nature. As we highlighted previously, tradition is not something that is frozen in time. We have seen that tradition embraces new behaviors, as long as it doesn’t lose its structure and central elements. These central elements are those that dialog with who we are as humans, with our biocultural body, our universal aspects. It is not a matter of culture, race, gender, social condition, age and so on. Fighting games, kites, and spinning tops are games that reflect this proximity and do not require words to bring different cultures together. This gestural proximity also facilitates the approximation with those games that we are not familiar with, and through these we also establish a dialog with the difference. The horizontal relations of the game allow for an appreciation of other perspectives.

The close relationship with the environment and the development of equipment with resources from nearby nature also fall in line with sustainability. TSG frequently uses organic resources, and care for the environment is fundamental to the possibilities of play. There is no need for equipment or highly elaborated spaces with external resources as games are organized according to the spaces and resources available, they also end without signs of depredation. During a serious environmental crisis that we are going through, this is an issue that has attracted the attention of sports researchers (Edgar, 2020). Children who participate in TSG festivals learn to share spaces and simple equipment. They also learn to make their own equipment from organic elements: wood, stones, ropes, straw, leather. It is important to notice that they wish to make this equipment, to work with their own hands, tools and materials as every human did before them. It seems to be important nowadays to establish clearer differences between having and being, and TSGs contribute to this inference.

The major sustainability issue for traditional communities is the achievement of sustainable management of species. This happens through the accurate observation of the environment, understanding it, the feeling of interdependence with the habitat, and empathy with an ecocentric vision. In traditional communities, it is common for children’s games to be exercises to improve skills and perceptions. In the Talking Circles, the researchers identified these same perceptual exercises at these festivals in the city.

Moreover, getting to know our body and how we play is to understand who we are, and it is very revealing and empowering for oneself. By playing games, we better understand our potential. Empowerment is fostered in a collective construction if we consider the possibility of an ethical debate from TSG practices. During Festivals, we saw children experiencing limits, possibilities and new elements. In a playful relationship, there is always the possibility of learning from the more experienced and helping the beginners. It is a dialogical teaching experience. Heeding the call of a game that attracts us is also going toward who we are.

Traditional Sports and Games promotes global health as it allows for diversity and dialog, the meeting between tradition and novelty. So, TSG Festivals have much to contribute to empowerment by facilitating accessibility to all levels of cultural diversity–schools, public spaces, networks and associations.

## Final Considerations

Traditional cultures and childhoods know the world through bodily and perceptual knowledge. This knowledge is created and recreated with imagination, logical reasoning, thought, and intimate relationship with the living world. It is also remembered in our body, updated in our gestures. Once embodied, this knowledge fosters respect, cooperation, and solidarity. Besides, considering the dialog with simple equipment and values from traditional communities, they bring relevant examples of respect for the environment. In its applicability, TSG proved to be important as an educational practice in public teaching programs, to present ways of inhabiting public spaces and promoting health, with the festive presence of human movement.

This article presents results from an initial study, and the topic further researches with different methodologies, which we hope to inspire. Beyond research, Festivals on TSG should be a theme to promote and look at from different perspectives. Play and movement had been associated with health not only by its objectives elements but also for its symbolic and significant ways of operating this body language and knowledge that we tried to highlight. We recommend and encourage Festivals and further research about it. Comparative elements could enrich and reinforce the results we found in this research. Although they seem initial, they sustain outcomes from many years of theoretical study and field observation.

By relating the concepts of sustainability, equality, and empowerment of traditional communities with events in urban centers, we believe it was possible to outstand how a TSG Festival may be invigorating to people in ecology, even in the city. In this sense, TSG reflects a way of life that we are invited to look at, especially nowadays, as it reveals and updates the fundamental images and values from these populations in an intercorporeal dialog. “Doing-together,” a widely found premise in traditional communities, stood out to promote intercultural dialog, sustainability and empowerment. This existential doing-together dialogs with traditional perceptual knowledge and happens without the need for words. This knowledge emphasizes the primacy of experience, gestural, and corporal reference.

Observations from a particular TSG Festival, in this case held in São Paulo, do not allow universalization of results. However, elements from this experience may indicate the potency of TSG. Through this study, we could verify how human gestures are updated in TSG, in the relationships established between community events and festivals in the city. Moreover, it is not only the gestures that are updated, as values, sustainable ways of thinking, and livings are present in this bodily repertoire. Empowerment happens in the body, under the “I can” premise. Considering that TSG is updated consistently in dialog with new generations, they also present the possibility to overcome barriers such as gender. This possibility creates a chance for learning how to solve conflicts. Playing together embraces us all. It is a profound and serious subject, although festive and playful. That is how TSG festivals operate: monitoring, updating, and keeping the challenges of producing humankind knowledge alive.

## Data Availability Statement

The raw data supporting the conclusions of this article will be made available by the authors, without undue reservation.

## Ethics Statement

All procedures followed were in accordance with the ethical standards fpr studies involving human subjects. The participants provided their written informed consent to participate in this study.

## Author Contributions

Both authors gave substantial contribution to the study conception and design, both being responsible for preparation and participation in the empirical work, data collection, conducting talking circles, analysis and writing of the manuscript, and approved the submitted version.

## Conflict of Interest

The authors declare that the research was conducted in the absence of any commercial or financial relationships that could be construed as a potential conflict of interest.
